# Supplementary material for: Repellency of Carvacrol, Thymol, and Their Acetates against Imported Fire Ants
Source: Insects. 2023 Sep 28;14(10):790. doi: 10.3390/insects14100790 (PMC10607101; doi:10.3390/insects14100790)
Supplement: Supplementary file 1 [file insects-14-00790-s001.zip › insects-2566355-supplementary.pdf]

Repellency of Carvacrol, Thymol, and Their Acetates against Imported Fire Ants

Pradeep Paudel[1], Farhan Mahmood Shah[1], Dileep Kumar Guddeti[1], Abbas Ali[1], Jian Chen[2], Ikhlas A. Khan[1,3], and Xing-Cong Li[1,3,*]

[1] National Center for Natural Products Research, School of Pharmacy, The University of Mississippi, University, MS 38677, USA; phr.paudel@gmail.com (P.P.); fshah@olemiss.edu (F.M.S.); gdileepkumar19@gmail.com (D.K.G.); aali@olemiss.edu (A.A.); ikhan@olemiss.edu (I.A.K.)

[2] Biological Control of Pests Research Unit, USDA-ARS, Stoneville, MS 38776, USA; jian.chen@usda.gov

[3]Department of BioMolecular Sciences, School of Pharmacy, The University of Mississippi, University, MS 38677, USA

*Correspondence: xcli7@olemiss.edu; Tel.: +1-662-915-6742

Legends

Figure S1: GC-MS profiles of (a) thyme and (b) red-thyme essential oils.

Figure S2: ^{1}H-NMR spectrum of carvacrol acetate in $CDCl_3$.

Figure S3: ^{13}C-NMR spectrum of carvacrol acetate in $CDCl_3$.

Figure S4: ^{1}H-NMR spectrum of thymol acetate in $CDCl_3$.

Figure S5: ^{13}C-NMR spectrum of thymol acetate in $CDCl_3$.

Table S1: List of compounds in Red-Thyme oil identified by GC-MS analysis.

Table S2: List of compounds in Thyme oil identified by GC-MS analysis.

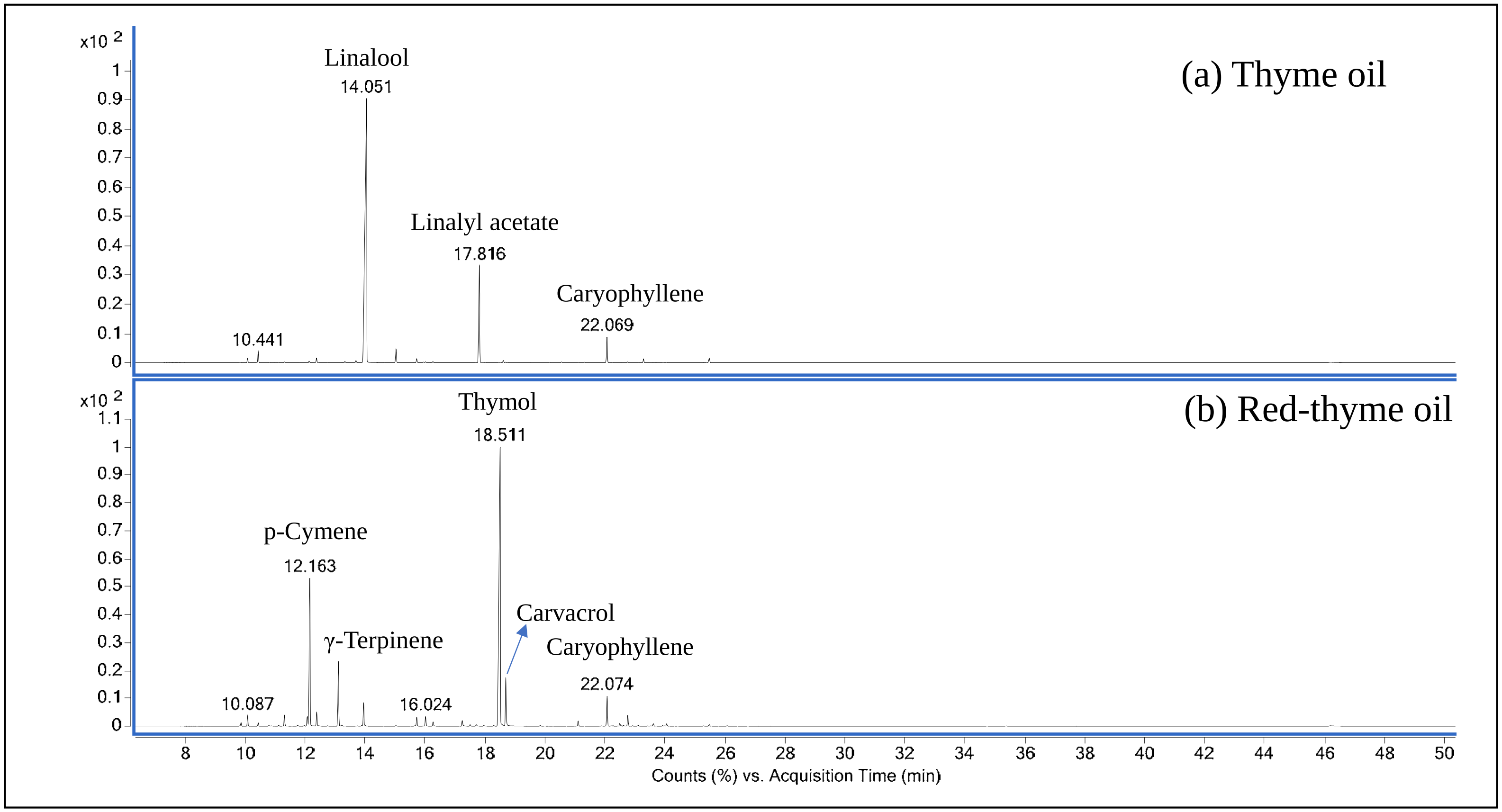

Figure S1: GC-MS profiles of (a) thyme and (b) red-thyme essential oils.

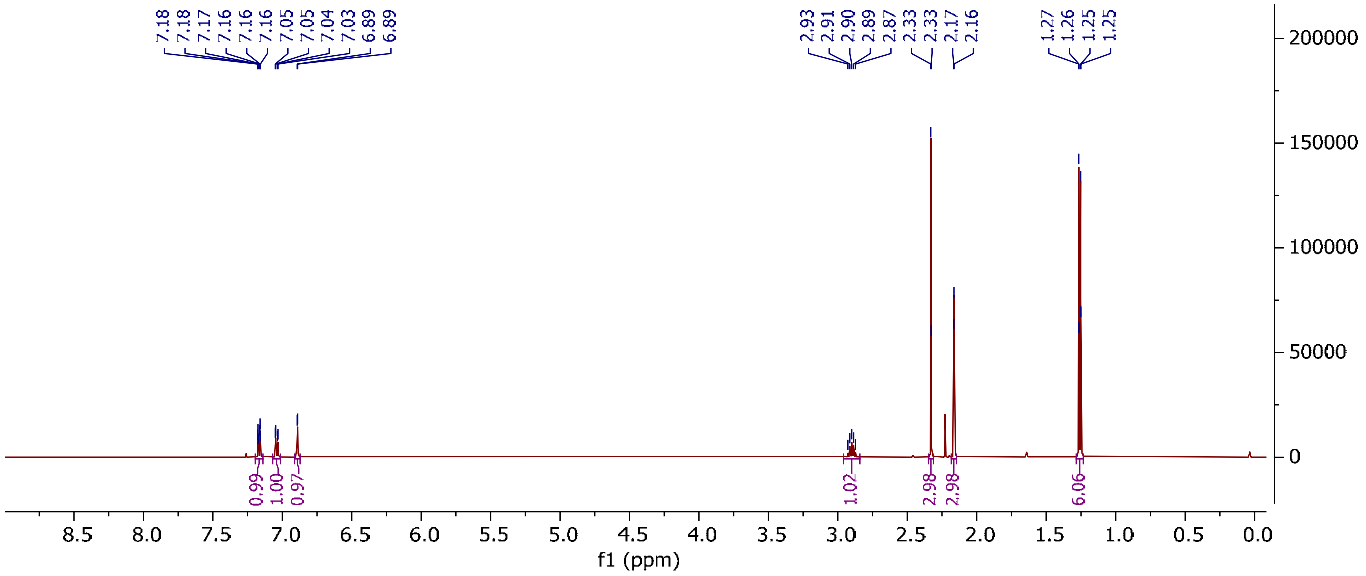

Figure S2: ^{1}H-NMR spectrum of carvacrol acetate in $CDCl_3$.

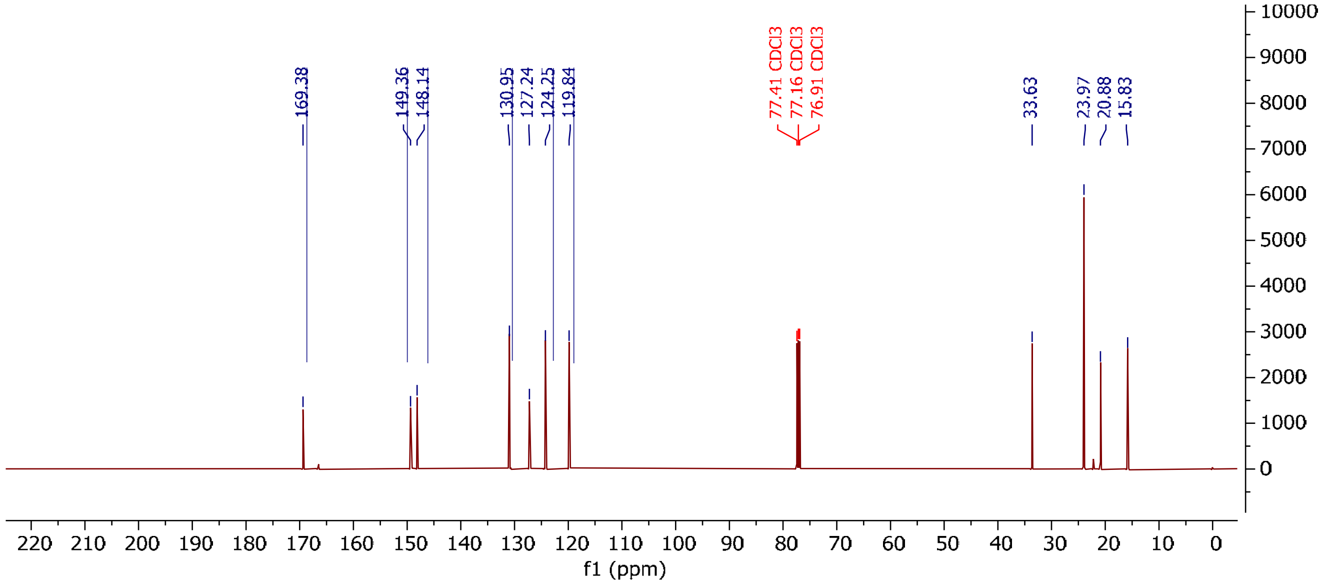

Figure S3: ^{13}C-NMR spectrum of carvacrol acetate in $CDCl_3$.

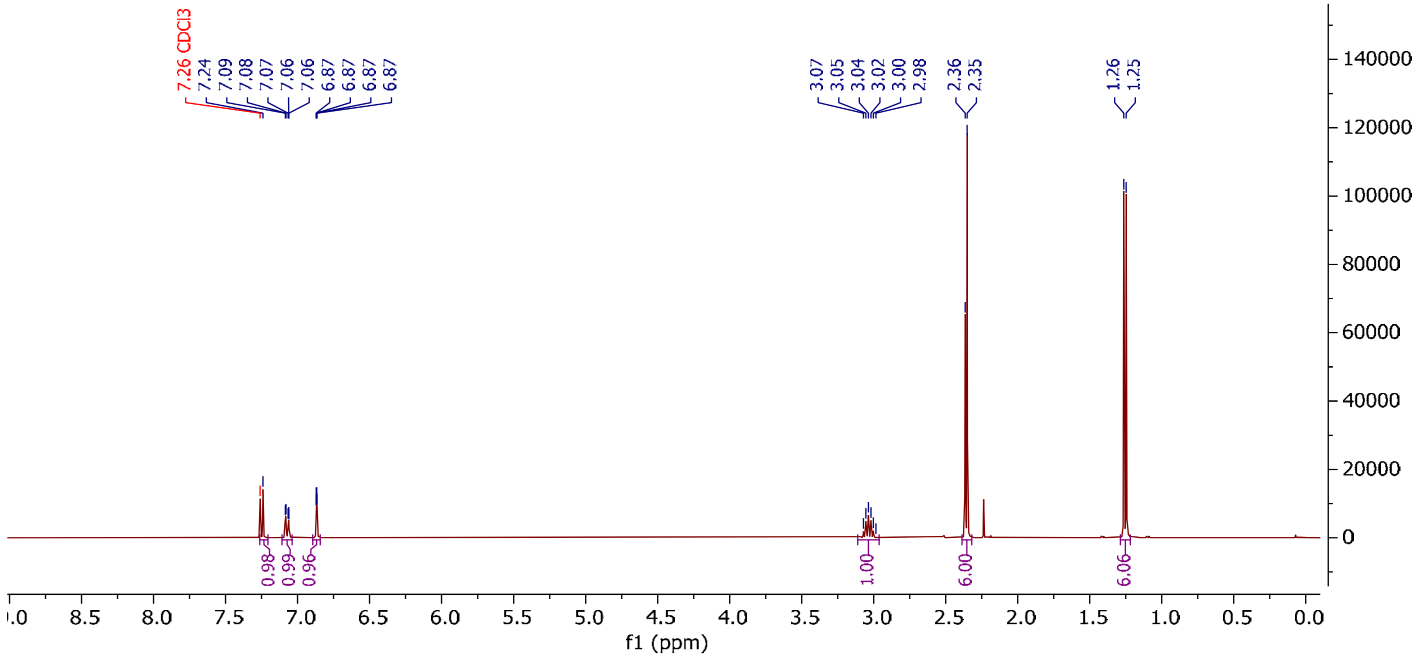

Figure S4: ^{1}H-NMR spectrum of thymol acetate in $CDCl_3$.

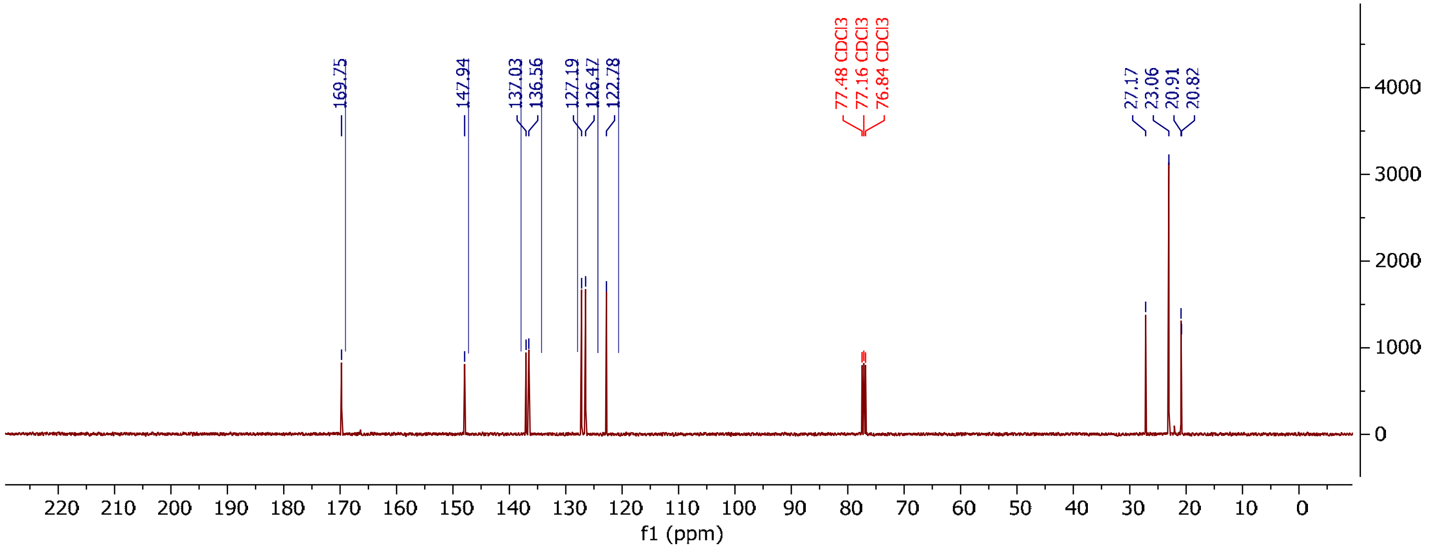

Figure S5: ^{13}C-NMR spectrum of thymol acetate in $CDCl_3$.

Table S1: List of compounds in Red-Thyme oil identified by GC-MS analysis.

S. NO	Retention time (min)	Compound	% Composition
1	9.869	α-Thujene	0.369
2	10.086	(+)-α-Pinene	0.958
3	10.442	Camphene	0.314
4	10.799	1-Octen-3-ol	0.080
5	10.879	3-Octanone	0.053
6	11.125	β-Pinene	0.108
7	11.312	β-Myrcene	1.074
8	11.756	α-Phellandrene	0.121
9	11.975	3-Carene	0.069
10	12.077	Terpinolene	1.011
11	12.163	p-Cymene	16.321
12	12.393	Eucalyptol	1.423
13	12.751	β-Ocimene	0.049
14	13.115	γ-Terpinene	6.418
15	13.232	β-Terpineol	0.140
16	13.954	Linalool	2.432
17	15.040	Camphor	0.076
18	15.725	(+)-Borneol	1.018
19	16.024	(-)-Terpinen-4-ol	1.049
20	16.270	Terpineol	0.528
21	17.244	O-Methylthymol	0.648
22	17.506	Methyl Carvacrol	0.169
23	17.709	Geraniol	0.215
24	17.955	Citral	0.143
25	18.511	Thymol	48.829
26	18.699	Carvacrol	5.100
27	19.854	4-Isopropylanisole	0.107
28	20.282	Carvacrol acetate	0.027
29	21.111	α-Copaene	0.528
30	21.303	β-Bourbonene	0.027
31	22.074	Caryophyllene	3.137
32	22.496	Aromandendrene	0.363
33	22.764	Humulene	1.152
34	22.929	Alloaromadendrene	0.056
35	23.122	gamma.-Muurolene	0.127
36	23.614	10s,11s-Himachala-3(12),4-diene	0.279
37	23.924	(+)-α-Muurolene	0.084
38	24.069	Cadina-1(10),4-diene	0.276
39	25.486	Caryophyllene oxide	0.202

Table S2: List of compounds in Thyme oil identified by GC-MS analysis.

S. NO	Retention time (min)	Compound	% Composition
1	10.088	(+)-α-Pinene	0.511
2	10.441	Camphene	1.325
3	11.125	β-Phellandrene	0.050
4	11.318	β-Myrcene	0.114
5	12.142	p-Cymene	0.227
6	12.387	Eucalyptol	0.583
7	13.336	cis-Linalool oxide (furanoid)	0.171
8	13.704	trans-Linalool oxide (furanoid)	0.299
9	14.051	Linalool	71.891
10	15.035	Camphor	1.704
11	15.727	(+)-Borneol	0.513
12	15.951	3,7-Octadiene-2,6-diol, 2,6-dimethyl-	0.110
13	16.022	(-)-Terpinen-4-ol	0.154
14	16.267	α-Terpineol	0.180
15	17.816	Linalyl acetate	13.386
16	18.615	Bornyl acetate	0.303
17	18.696	Carvacrol	0.145
18	20.162	Geranyl palmitate	0.070
19	20.554	Geranyl acetate	0.111
20	21.309	β-Bourbonene	0.090
21	22.069	Caryophyllene	3.385
22	22.272	α-trans-Bergamotene	0.054
23	22.766	Humulene	0.105
24	23.290	Germacrene D	0.472
25	24.064	Cadina-1(10),4-diene	0.052
26	25.481	Caryophyllene oxide	0.716
